# Supplementary material for: Improved Outcomes in Myelofibrosis after Allogeneic Stem-Cell Transplantation in the Era of Ruxolitinib Pretreatment and Intensified Conditioning Regimen—Single-Center Analysis
Source: Cancers (Basel). 2024 Sep 25;16(19):3257. doi: 10.3390/cancers16193257 (PMC11482566; doi:10.3390/cancers16193257)
Supplement: Supplementary file 1 [file cancers-16-03257-s001.zip › cancers-3174024-supplementary.pdf]

Parameters included in the univariate and multivariate analysis:

Gender (male / female); CMV status recipient (CMV positive / CMV negative); Age at HSCT (years): <55 years, 55 years; CD34 x106/kg body weight recipient; Stem cell source (peripheral blood / bone marrow); Conditioning regimen (MAC/RTC / RIC/NMA); Donor type, dichotomous (MRD / other); GVHD prophylaxis, dichotomous (CSA/MMF / CSA/MTX or PTCy/Tac/MMF); Osteosclerosis at HSCT, dichotomous (grade 0+1 / grade 2+3); Reticulin fibrosis bone marrow prior to HSCT, dichotomous (grade 0+1 / grade 2+3); Diagnosis at the time of diagnosis, dichotomous [PMF or MDS/MPN or sec. MF/ Excess of blasts (10-<20%)/ blast crisis (≥20%)]; State of disease at HSCT, dichotomous [chronic (blasts < 10%) / accelerated (10−19% blasts) / secondary leukemia (≥20% blasts)]; RBC transfusion dependency prior to HSCT (≥2 RBC transfusions per month for at least three months prior to HSCT) (yes / no); PLT transfusion dependency prior to HSCT (≥1 PLT transfusion per month for at least three months prior to HSCT) (yes / no); Gender match (female donor to male recipient / other combination); aGVHD (2-4,3-4) (yes / no); cGVHD, dichotomous (no cGVHD+limited / extensive); Ruxolitinib 3 months prior to HSCT (yes / no); Interval transplantation date to latest transplantation date (days); spleen size at transplantation (≥22cm)(yes/no); HCT-CI (3 / <3);
Driver mutations at diagnosis, dichotomous (JAK2 + CALR / MPL + TN); Non driver mutations (≥3) at transplantation (yes / no); high risk mutations at transplantation (ASXL1, SRSF2, EZH2, IDH1/IDH2 or U2AF1) (yes / no); DIPSS at HSCT, dichotomous (low risk + intermediate-1 risk / intermediate-2 risk + high risk); DIPSS plus at transplantation, dichotomous (low risk + intermediate-1 risk / intermediate-2 risk + high risk); MIPSS 70 plus at HSCT, dichotomous (low risk + intermediate risk / high risk + very high risk); MTSS at transplantation, dichotomous (low risk + intermediate risk / high risk + very high risk); Cytogenetics at diagnosis, dichotomous (normal or other / unfavorable)

Supplemental Table S1: Causes of death

UPN	TxDate	Age	Gender	State of Disease at Tx	HCT-CI	RUX before Tx	DIPPS+ Tx	MTSS Tx	MIPSS+ Tx	Donor Type	Conditioning intensity	Conditioning regimen	OS from Tx (months)	Cause of death
22	13.03.2001	66	m	CP	2	no		high		MRD	RIC/NMA	FB2	1	fungal infection/sinus cavernosus thrombosis

33	18.07.2003	51	f	CP	3	no		int		MRD	RIC/NMA	FB2	1	fungal infection/multiorgan failure
8	13.07.2004	63	m	CP	0	no	int 2	low	very high	MRD	RIC/NMA	FB2	92	relapse
31	07.06.2006	43	f	BP	0	no		high		MMUD	RIC/NMA	FB2-ATG (20)	49	chronic GvHD/pneumonia
24	28.04.2009	59	m	CP	0	no	int 2	low	very high	MUD	RIC/NMA	FB2-ATG (20)	4	acute GvHD
40	26.01.2010	54	m	CP	1	no	high	low	very high	MRD	RIC/NMA	FB2	7	acute GvHd/ pneumonia
6	17.11.2011	52	m	CP	0	no	int 2	int	high	MUD	RIC/NMA	FB2-ATG (20)	102	accident
17	05.06.2012	63	m	CP	2	no	high	high	very high	MUD	RIC/NMA	FB2ATG(20)	76	second primary malignancy
2	25.06.2012	63	m	CP	1	no	int 2	low	high	MRD	RIC/NMA	FB2	3	acute GvHD
7	04.09.2012	66	f	CP	2	no	high	very high		MMUD	RIC/NMA	FB2-ATG (20)	2	acute GvHD
19	22.03.2013	58	m	CP	3	no	int 2			MMUD	RIC/NMA	FB2-ATG (20)	7	acute GvHD
25	27.10.2016	63	m	BP	4	no	int 1	int	very high	HAPLO	MAC/RTC	Cy-FB3	2	infection/ septicaemia
3	21.12.2016	63	m	CP	0	yes	high	high	very high	MUD	MAC/RTC	FLAC-FB3-ATG(40)	3	acute GvHD
38	14.03.2018	40	f	CP	1	yes	int 1	low	high	MRD	MAC/RTC	FLAC-FB3-ATG(30)	22	relapse, pneumonia
34	11.03.2021	50	m	AP	5	no	int 1	high	very high	MRD	RIC/NMA	FLAC-ATG(35)-TBI 4Gy	1	pneumonia

CP= chronic phase, BP= blast phase, MRD= matched related donor, MUD= matched unrelated donor, MMUD= mismatched unrelated donor, HAPLO= haploidentical (related) donor, RIC= reduced intensity conditioning, NMA= non-myeloablative, MAC= myeloablative conditioning, RTC= reduced toxicity conditioning, GvHD= graft versus host disease

Supplemental Table S2:

Kaplan-Meier-Estimation (competing risk estimation) of overall survival, progression-free survival, cumulative incidence of relapse and cumulative incidence of non-relapse mortality

	**3 year OS (%) [95% CI]**	**3 year PFS (%) [95% CI]**	**3 year cumulative incidence of relapse (%) [95% CI]**	**3 year cumulative incidence of non relapse mortality (%) [95% CI]**
Whole cohort	68.9% [55.2% - 86.1%]	65.4% [51.0% - 83.7%]	5.6% [0% - 21.0%]	27.8% [11.6% - 41.0%]
Until 31.12.2015	53.3% [33.2% - 85.6%]	44.4% [24.6% - 80.5%]	6.7% [0% - 41.7%]	46.7% [14.4% - 66.8%]
From 01.01.2016	79.6% [63.4% - 100%]	81.0% [65.8% - 99.6%]	4.8% [0% - 15.6%]	14.3% [0% - 28.0%]
from 2016 with TBI	87.5% [67.3% - 100%]	87.5% [67.3% - 100%]	0.0% [§]	12.5% [0% - 32.7%]
from 2016 without TBI	76.9% [57.1% - 100%]	76.9% [57.1% - 100%]	7.7% [0% - 24.6%]	15.4% [0% - 32.9%]
from 2016 with sequential conditioning	78.7% [59.7% - 100%]	81.3% [64.2% - 100%]	6.3% [0% - 19.7%]	12.5% [0% - 27.3%]
from 2016 without sequential conditioning	80.0% [51.6% - 100%]	80.0% [51.6% - 100%]	0.0% [§]	20.0% [0% - 48.4%]

Supplemental Table S3:
Logistic regression analysis of changes in reticulin fibrosis

		Reticulin fibrosis improvement at 12 months compared to Tx					
		0 + 1 points		2 + 3 points		Total	
		n	%	*n*	%	*n*	%
cGvHD	no cGVHD + limited	6	100.00	2	25.00	8	57.14
	extensive	0	0.00	6	75.00	6	42.86
	Total	6	100.00	8	100.00	14	100.00

Tx timepoint of transplantation

Supplemental Table S4:
Logistic regression analysis of changes in spleen length

		Spleen length at 12 months compared to Tx					
		decrease > 25%		unchanged		Total	
		n	%	n	%	n	%
Non- driver mutations ≥ 3 at Tx	no	3	37.50	8	100.00	11	68.75
	yes	5	62.50	0	0.00	5	31.25
	Total	8	100.00	8	100.00	16	100.00

Tx timepoint of transplantation

Supplemental Table S5:
Univariate analysis (complete data)

Parameter	Reticulin fibrosis improvement at 6Mo compared to Tx (0 or 1 versus 2 or 3 points)		Reticulin fibrosis improvement at 12Mo compared to Tx (0 or 1 versus 2 or 3 points)		Spleen length at 6Mo compared to Tx (>25%/unchanged)		Spleen length at 12Mo compared to Tx (>25%/unchanged)	
	**Test**	***p*-Wert**	**Test**	***p*-Wert**	**Test**	***p*-Wert**	**Test**	***p*-Wert**
CD34 x10'6 / kg BW recepient	MWU	0.401	MWU	0.142	MWU	>0.999	MWU	0.959
Age at Dx (days)	t-test	0.441	t-test	0.408	t-test	0.356	t-test	0.578
Interval Tx Date to latest Tx Date (days)	t-test	0.463	MWU	0.108	MWU	0.482	MWU	0.878
Sex	Fisher	0.615	Fisher	>0.999	Fisher	>0.999	Fisher	0.119
Spleen at Tx ≥ 22 cm	Fisher	0.083	Fisher	>0.999	Fisher	>0.999	Fisher	>0.999
HCT-CI (cat.)	Fisher	>0.999	Fisher	>0.999	Fisher	>0.999	Fisher	0.467
Non driver mutations ≥ 3 at Tx	Fisher	0.245	Fisher	>0.999	Fisher	>0.999	Fisher	**0.026***
HMR at Tx ≥ 1	Fisher	>0.999	Fisher	0.592	Fisher	0.646	Fisher	0.119
Ruxolotinib ≥ 3 months prior to Tx	Fisher	0.099	Fisher	0.580	Fisher	0.649	Fisher	>0.999
RBC transfusion dependecy prior to Tx	Fisher	0.608	Fisher	>0.999	Fisher	0.351	Fisher	>0.999
PLT transfusion dependency prior to Tx	Fisher	0.490	Fisher	>0.999	Fisher	0.364	Fisher	>0.999
Stemcell source	Fisher	>0.999	Fisher	>0.999	Fisher	>0.999	Fisher	>0.999
Conditioning regimen	Fisher	>0.999	Fisher	0.103	Fisher	>0.999	Fisher	0.619

CMV status recipient	Fisher	>0.999	Fisher	0.301	Fisher	0.187	Fisher	>0.999
Sex match (donor/ recepient)	Fisher	>0.999	Fisher	0.627	Fisher	>0.999	Fisher	0.315
aGVHD (2-4.3-4)	Fisher	0.615	Fisher	>0.999	Fisher	>0.999	Fisher	>0.999
Retikulinfibrosis BM prior Tx grade [dichotomously.]	Fisher	>0.999	Fisher	0.165	Fisher	>0.999	Fisher	0.417
Osteosclerosis grade [dichotomously]	Fisher	>0.999	Fisher	>0.999	Fisher	0.131	Fisher	>0.999
DIPSS score at Tx [dichotomously]	Fisher	>0.999	Fisher	0.580	Fisher	0.662	Fisher	>0.999
DIPSS+ score at Tx [dichotomously]	Fisher	>0.999	Fisher	>0.999	Fisher	0.389	Fisher	>0.999
MIPSS70+ at Tx [dichotomously]	Fisher	0.515	Fisher	>0.999	Fisher	0.560	Fisher	>0.999
MTSS score at Tx [dichotomously]	Fisher	>0.999	Fisher	0.293	Fisher	0.651	Fisher	>0.999
cGvHD [dichotomously]	Fisher	>0.999	Fisher	**0.010***	Fisher	>0.999	Fisher	>0.999
Diagnosis at Dx in 3 groups [dichotomously]	Fisher	>0.999	Fisher	>0.999	Fisher	>0.999	Fisher	>0.999
Driver mutations [dichotomously]	Fisher	0.490	Fisher	0.473	Fisher	>0.999	Fisher	0.467
Disease status at Tx [dichotomously]	Fisher	>0.999	Fisher	>0.999	Fisher	>0.999	Fisher	>0.999
Cytogenetics at Dx + - 12 months [dichotomously]	Fisher	>0.999	Fisher	>0.999	Fisher	>0.999	Fisher	>0.999
Donor type [dichotomously]	Fisher	0.615	Fisher	0.580	Fisher	0.343	Fisher	0.315
GVHD prophylaxis [dichotomously]	Fisher	0.326	Fisher	0.592	Fisher	0.351	Fisher	0.608

Tx = timepoint of transplantation, Dx = timepoint of diagnosis, * *p*-values with significance bold

Supplemental Table S6:
Cox regressions
Overall survival

Multivariate model	Regression coefficient B	*p*-value	Hazard-Ratio	95% Confidence interval Hazard-ratio	
				Lower limit	Upper limit
Age at Dx	-0.002	0.961	0.998	0.937	1.063
Interval Tx date to latest Tx Date	0.000	0.003*	1.00040	1.00013	1.00067

Tx = timepoint of transplantation, Dx = timepoint of diagnosis

Supplemental Table S7:

Cox regressions
Relapse

Multivariate Model	Regression coefficient B	*p*-value	Hazard-ratio	95% Confidence interval Hazard-ratio	
				Lower limit	Upper limit
CD34 x10'6 / kg BW recipient	0.548	0.391	1.730	0.494	6.058
Age at Dx	-0.096	0.319	0.908	0.751	1.098
Interval Tx date to latest Tx date	0.001	0.191	1.001	0.9996	1.0019

Tx = timepoint of transplantation, Dx = timepoint of diagnosis, BW = body weight

Supplemental Table S8:

Cox regressions

Non relapse mortality

Multivariate model	Regression coefficient B	*p*-value	Hazard-ratio	95% Confidence interval Hazard-ratio	
				Lower limit	Upper limit
Age at Dx	0.015	0.677	1.01498	0.94641	1.08851
Interval Tx date to latest Tx date	0.000	**0.004***	1.00042	1.00014	1.00070

Tx = timepoint of transplantation, Dx = timepoint of diagnosis, * *p*-values with significance bold

Supplemental Table S9:

Cox regressions

Progression free survival

Multivariate model	Regression coefficient B	*p*-value	Hazard-ratio	95% Confidence interval Hazard-ratio	
				Lower limit	Upper limit
Age at Dx	0.006	0.849	1.006	0.944	1.073
Interval Tx date to latest Tx date	0.000465	**0.001***	1.000465	1.000192	1.000738

Tx = timepoint of transplantation, Dx = timepoint of diagnosis, * *p*-values with significance bold

Supplemental Table S10:

Cox regressions

Time to neutrophil engraftment (ANC > 0.5 G/L)

Multivariate model	Regression coefficient B	*p*-value	Hazard-ratio	95% Confidence interval Hazard-ratio	
				Lower limit	Upper limit
Age at Dx	-0.003	0.856	0.997	0.960	1.034
Interval Tx date to latest Tx date	0.000	0.370	0.999903	0.999693	1.000114

Tx = timepoint of transplantation. Dx = timepoint of diagnosis

Supplemental Table S11:

Cox regressions

Time to platelet engraftment (PLT > 20 G/L)

Multivariate model	Regression coefficient B	*p*-value	Hazard-ratio	95% confidence interval Hazard-ratio	
				Lower limit	Upper limit
CD34 x10'6 / kg BW recipient	0.170	0.103	1.185	0.966	1.453
Age at Dx	0.005	0.808	1.005	0.962	1.050
Interval Tx Date to latest Tx Date	0.00009	0.430	1.00009	0.9999	1.0003

Tx = timepoint of transplantation. Dx = timepoint of diagnosis. BW = body weight

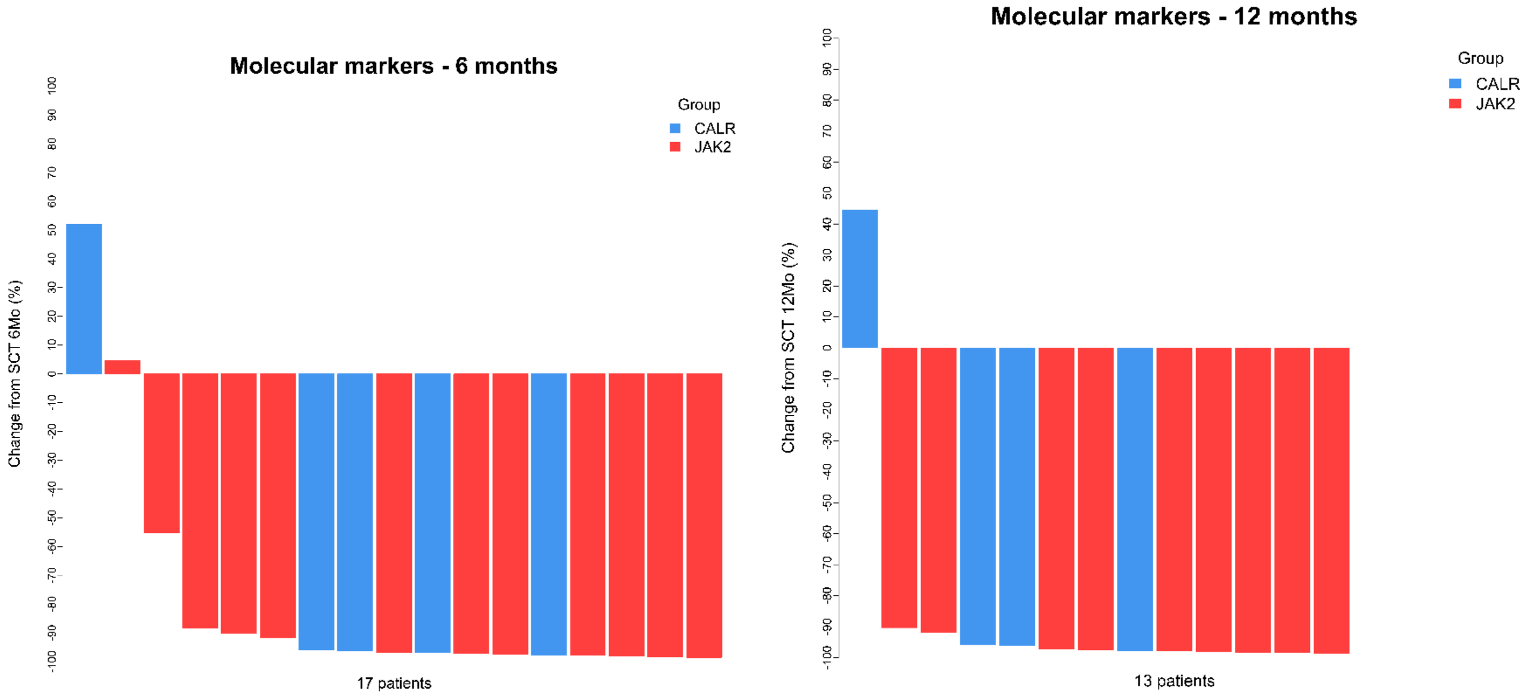

(a) (b)

Supplemental Figure S1 a-b: molecular response after 6 and after 12 months

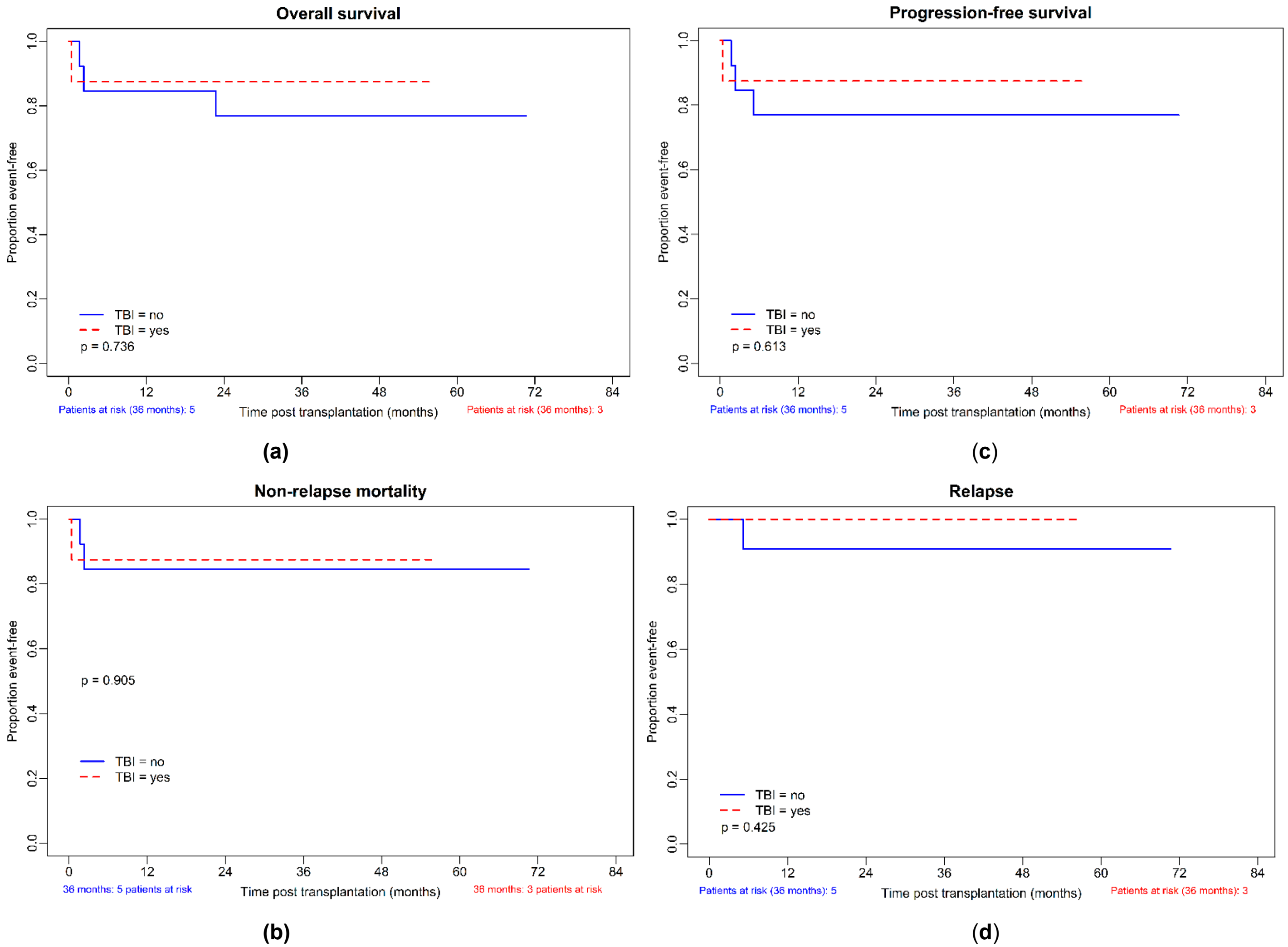

Supplemental Figure S2 a-d: OS, NRM, PFS and CIR in the patient cohort with alloHSCT after 01.01.206 (*n* = 21) comparing TBI-containing conditioning (n=8) versus non-TBI-containing conditioning (*n* = 13) regimen

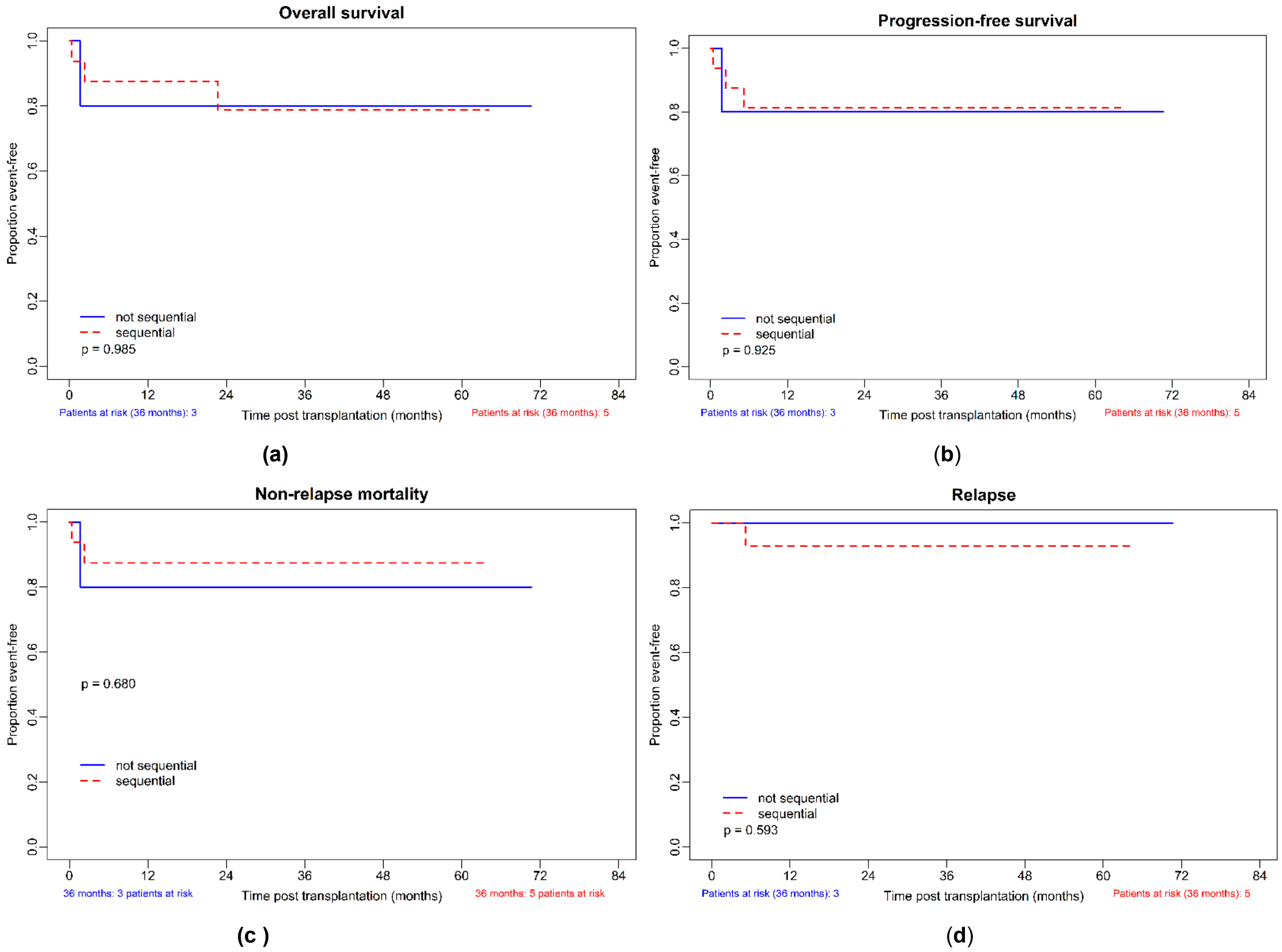
Overall survival
Proportion event-free
not sequential
sequential
p = 0.985
Time post transplantation (months)
Patients at risk (36 months): 3
Patients at risk (36 months): 5
(a)
Progression-free survival
Proportion event-free
not sequential
sequential
p = 0.925
Time post transplantation (months)
Patients at risk (36 months): 3
Patients at risk (36 months): 5
(b)
Non-relapse mortality
Proportion event-free
p = 0.680
not sequential
sequential
Time post transplantation (months)
36 months: 3 patients at risk
36 months: 5 patients at risk
(c)
Relapse
Proportion event-free
not sequential
sequential
p = 0.593
Time post transplantation (months)
Patients at risk (36 months): 3
Patients at risk (36 months): 5
(d)

Supplemental Figure S3 a-d: OS, PFS, NRM and CIR in the cohort of patients with alloHSCT after 01.01.2016 (*n* = 21) comparing sequential conditioning (*n* = 16) versus non sequential conditioning (*n* = 5) regimen
